# *Leishmania major* Methionine Sulfoxide Reductase A Is Required for Resistance to Oxidative Stress and Efficient Replication in Macrophages

**DOI:** 10.1371/journal.pone.0056064

**Published:** 2013-02-20

**Authors:** Fiona M. Sansom, Leonie Tang, Julie E. Ralton, Eleanor C. Saunders, Thomas Naderer, Malcolm J. McConville

**Affiliations:** 1 Department of Biochemistry and Molecular Biology, Bio21 Institute of Molecular Science and Biotechnology, University of Melbourne, Parkville, Victoria, Australia; 2 Faculty of Veterinary Science, University of Melbourne, Parkville, Victoria, Australia; Louisiana State University, United States of America

## Abstract

*Leishmania* are protozoan parasites that proliferate within the phagolysome of mammalian macrophages. While a number of anti-oxidant systems in these parasites have been shown to protect against endogenous as well as host-generated reactive oxygen species, the potential role of enzymes involved in the repair of oxidatively damaged proteins remains uncharacterized. The *Leishmania* spp genomes encode a single putative methionine sulfoxide reductase (MsrA) that could have a role in reducing oxidized free and proteinogenic methionine residues. A GFP-fusion of *L. major* MsrA was shown to have a cytoplasmic localization by immunofluorescence microscopy and subcellular fractionation. An *L. major msrA* null mutant, generated by targeted replacement of both chromosomal allelles, was viable in rich medium but was unable to reduce exogenous methionine sulfoxide when cultivated in the presence of this amino acid, indicating that *msrA* encodes a functional MsrA. The Δ*msrA* mutant exhibited increased sensitivity to H_2_O_2_ compared to wild type parasites and was unable to proliferate normally in macrophages. Wild type sensitivity to H_2_O_2_ and infectivity in macrophages was restored by complementation of the mutant with a plasmid encoding MsrA. Unexpectedly, the Δ*msrA* mutant was able to induce normal lesions in susceptible BALB/c indicating that this protein is not essential for pathogenesis *in vivo*. Our results suggest that *Leishmania* MsrA contributes to the anti-oxidative defences of these parasites, but that complementary oxidative defence mechansims are up-regulated in lesion amastigotes.

## Introduction

*Leishmania* parasites are sand fly-transmitted protozoa that cause a spectrum of diseases in humans, ranging from localized cutaneous lesions to disseminating mucocutaneous and visceral infections [1]. More than 350 million people are at risk of infection worldwide, with an estimated 1.5 to 2 million new cases per year. Visceral leishmaniasis is associated with high mortality and an estimated 50,000 deaths each year [2]. While there have been recent advances in treatment options, current front line anti-leishmania therapies are limited by their high cost, limited availability and/or toxicity and the widespread resistance in endemic areas [3].

*Leishmania* develop as extracellular promastigotes in the mid-gut of the Phlebotomine sand fly vector. Non-dividing metacyclic promastigotes that accumulate in the sandfly mouthparts are injected into the mammalian host when infected, female sandflies take a blood meal and are phagocytosed by host macrophages, either directly or after passage through neutrophils [4]. Phagocytosed promastigotes are delivered to the mature phagolysosome compartment of macrophages where they differentiate to the non-motile, obligate intracellular amastigote stage [5]. Both developmental stages are likely to be exposed to oxidative stresses. In the sandfly mid-gut, reactive oxygen species (ROS) are likely to be generated during the digestion of blood hemoglobin, while in the mammalian host, ROS are generated during parasite invasion of naïve macrophages and in immune-activated host cells. *Leishmania* combat these stresses via a number of different and well-studied mechanisms. For example, invading promastigotes and amastigotes have been shown to actively inhibit recruitment of the NADPH oxidase complex to the phagolysosome membrane and to express a number of surface glycoconjugates and intracellular proteins that either scavenge extracellular and/or neutralize intracellular ROS species [6]–[8]. The latter include multiple isoforms of superoxide dismutase [9], ascorbate peroxidase [10]–[12], pteridine reductase [13], [14] as well as enzymes involved in the synthesis and regulation of the major cellular thiols, trypanothione and ovathiol [6], [15]–[17]. Genetic disruption of most of these enzymes leads to partial or complete attenuation of parasite growth and survival in the mammalian host [18]–[21].

In contrast to the mechanisms listed above, nothing is known about oxidative defense mechanisms that involve the repair of damaged proteins. Methionine, a sulfur-containing amino acid, is particularly vulnerable to oxidative damage and, in organisms such as yeast and *E. coli,* mechanisms exist to reverse oxidized methionine. The product of methionine oxidation, methionine sulfoxide (MetO), exists as two epimers, methionine-(S)-sulfoxide (Met-S-O) and methionine-(R)-sulfoxide (Met-R-O) [22], and three separate repair enzymes, known as methionine sulfoxide reductases (Msr) have been identified. Methionine sulfoxide reductase A (MsrA) acts on both free and protein-bound Met-S-O [23], methionine sulfoxide reductase B (MsrB) primarily repairs protein-bound Met-R-O [24] and methionine sulfoxide reductase C (MsrC) is able to reduce free Met-R-O [25], [26]. Of these enzymes MsrA is perhaps the best characterized, and is involved in resistance to oxidative stress and virulence for a number of bacteria including *Salmonella* [27], *Enterococcus faecalis* [28] and *Mycoplasma genitalium* [29], [30]. The related trypanosomatid parasite, *Trypanosoma cruzi*, has recently been shown to express a functional MsrA that confers increased resistance to oxidative stress when overexpressed in pathogenic stages [31]. However, the potential role of MsrA enzymes in the pathogenesis of *T. cruzi* or other protozoan parasites remains unknown. Here, we provide evidence that *L. major* encodes a functional MsrA and have investigated its role in resistance to oxidative stress, intracellular replication and parasite virulence within a mammalian host.

## Results

### Identification of the *Leishmania* Gene Encoding a Putative MsrA Enzyme with a Non- Classical Catalytic Site Sequence

**Figure 1:**
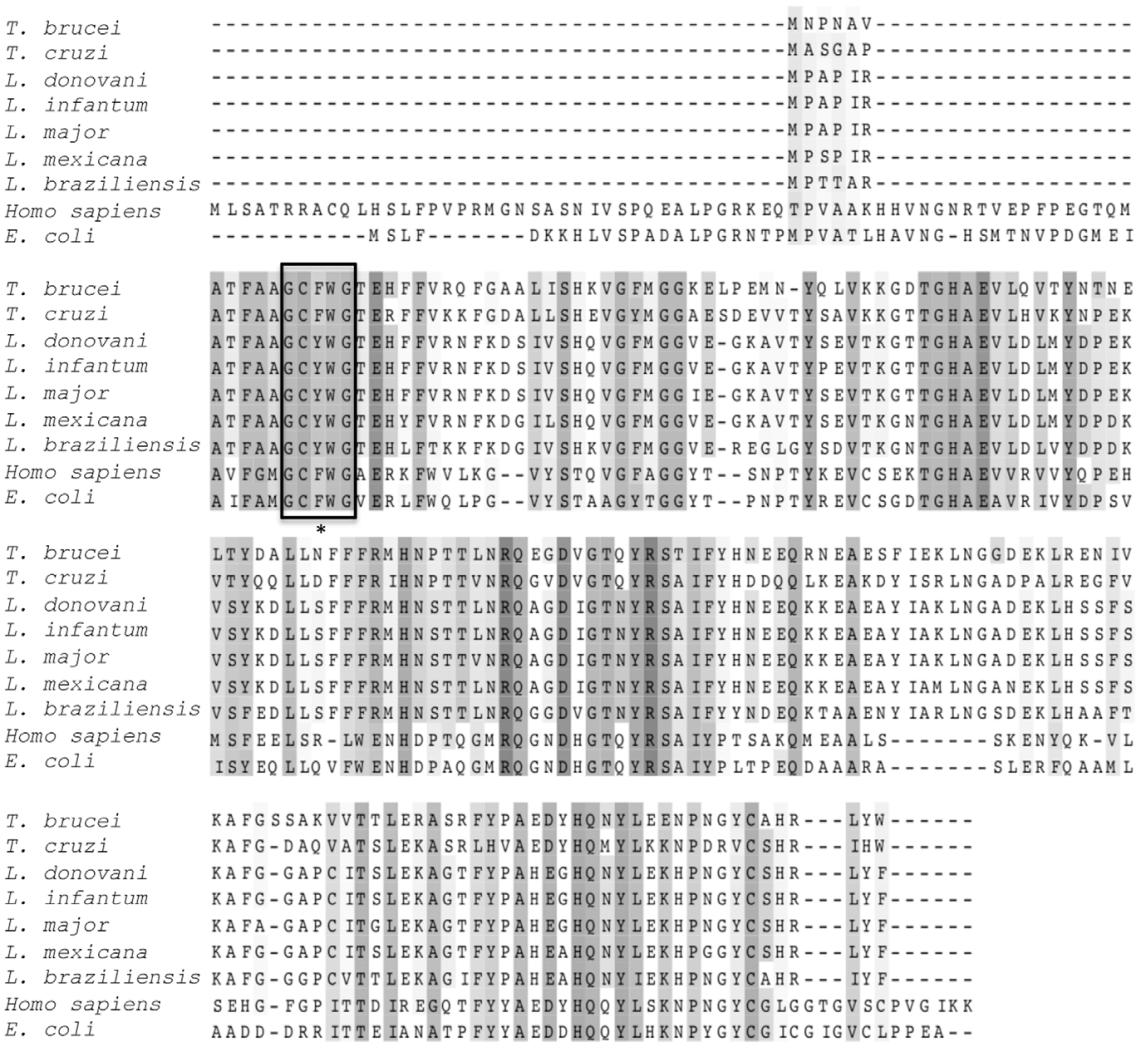
Sequence alignment of *L. major* MsrA with known and putative MsrA enzymes from other *Leishmania* species and *Trypanosoma* species, as well as human and *E. coli* MsrA. The catalytic site is boxed, and the non-conserved residue within the site marked by an *. The degree of amino acid conservation is indicated by shading intensity. Sequence alignment was performed using ClustalW [55], [56] and the resulting alignment viewed and edited using JalView software [57]. The following sequence accession numbers were used: *L. major* XP_001681026.1, L. *donovani* CBZ31842.1, *L. infantum* XP_001463331.1, *L. mexicana* CBZ23737.1, *L. braziliensis* XP_001562615.1, *T. brucei* XP_846892.1, *T. cruzi* XP_814806.1, *Homo sapiens* AAG09689.1, *E. coli* YP_001746621.1.

Analysis of the *L. major* strain Friedlin genome with the NCBI BLAST suite of programs [32] revealed the presence of a single open reading frame, *LmjF07.1140*, encoding a protein with 40% identity to human MsrA and 38% identity to MsrA from *E. coli.* Based on the analyses described below, we refer to this gene as *msrA* and the encoded protein as LmMsrA. Alignment of the predicted amino acid sequence with putative and known MsrA enzymes from other trypanosomatids revealed that LmMsrA is highly conserved across different species of *Leishmania* with greater than 90% identity between *L. major*, *L. infantum, L. donovani* and *L. mexicana* MsrA homologues (Figure 1). The sequence similarity with the more evolutionary divergent *Leishmania* species, *L. braziliensis,* was 76%, and lower identity (59–64%) was observed to MsrA enzymes from the related *Trypanosoma* parasite species. Intriguingly, all of the *Leishmania* MsrA sequences contained a single amino acid substitution within the catalytic site, in which a tyrosine residue that has been shown to be critical for enzymatic activity in other eukaryotic and bacterial MsrA [33] was replaced with a phenylalanine residue (GC**Y**WG to GC**F**WG) (Figure 1). This sequence change was not observed in the *T. cruzi* or *T. brucei msrA* homologues. Genes encoding a putative MsrB enzyme (*LmjF28_2660*) and a putative MsrC (*LmjF23_1460*) enzyme, with 37% and 39% identity to *E. coli* MsrB and MsrC, respectively, were also identified in these analyses. As studies in other microbial pathogens have highlighted the role of MsrA in pathogenesis [27], [34], [35], we focused our studies on defining the potential role of this enzyme in *L. major*.

### MsrA of *L. major* is Localized to the Cytoplasm of the Parasite and is not Essential for Parasite Viability

**Figure 2:**
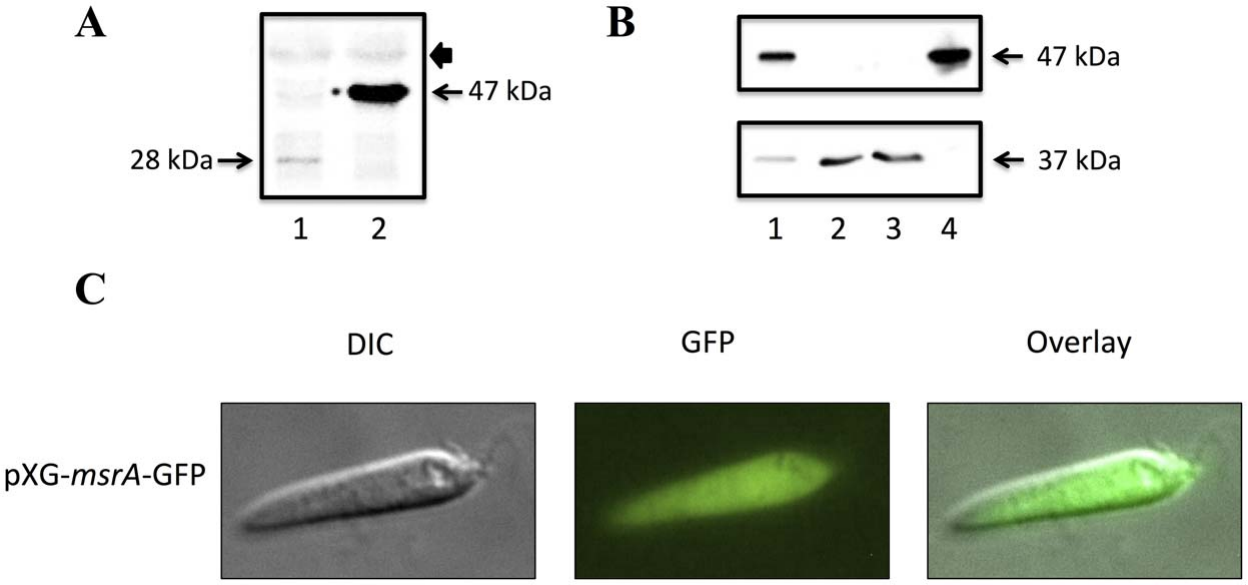
Subcellular localization of MsrA-GFP. A. Whole cell lysates of *L. major* transfected with pXG-‘GFP alone (Lane 1) or with pXG-*msrA*-GFP (lane 2) were analyzed by SDS-PAGE and proteins detected by Western blot using the anti-GFP antibody. Approximate molecular weights are as indicated. Cells transfected with pXG-‘GFP alone express a 28 kDa band corresponding to GFP alone, whereas cells transfected with pXG-*msrA*-GFP express the 47 kDa MsrA-GFP fusion protein. Similar non-specific cross reaction with a higher molecular weight band (arrow head) indicates equal sample loading. B. *L. major* promastigotes transfected with pXG-*msrA*-GFP were lysed and subjected to differential centrifugation. Fractions were probed with anti-GFP (top panel), then reprobed with anti-FBP (bottom panel) to confirm the integrity of organelles following cell lysis. Lane 1, whole cell lysate; Lane 2, 10,000*×g* organelle fraction; Lane 3, 100,000*×g* microsomal fraction; Lane 4, supernatant (cytosolic) fraction. LmMsrA-GFP is present only in whole cells and the cytosol. The glycosome enzyme, FBP, was only detected in the total extract and in the organelle and microsomal fractions confirming the integrity of the organellar fraction. C. Epifluroscent microscopy images of *L. major* promastigotes infected with pXG-*msrA*-GFP constructs. Cytoplasmic green fluorescence is seen in the parasites expressing the LmMsrA-GFP fusion protein.

MsrA has been localized to both the cytosol and organellar compartments of eukaryotic cells [36], [37]. The LmMsrA amino acid sequence lacks conspicuous organelle targeting signals, indicating a possible cytoplasmic localization. To confirm the localization of this protein, an LmMsrA-GFP fusion protein was expressed in *L. major* promastigotes. As expected, episomal expression resulted in production of a 47 kDa protein, the expected size, as detected by Western blotting using an anti-GFP antibody (Figure 2A). No GFP breakdown by-product was observed in the cells expressing pXG-*msrA*-GFP. Consistent with the lack of targeting signals, epifluorescent microscopy of live parasites revealed a cytosolic location for LmMsrA, with bright green fluorescence distributed evenly through the cytoplasm (Figure 2C). To confirm the absence of low level organellar targeting that might be masked by the strong cytoplasmic localization, *L. major* promastigotes expressing the fusion protein were lyzed and organellar fractions separated from cytosol by differentiation centrifugation. As shown in Figure 2B, full length LmMsrA-GFP was recovered in the cytosolic fraction. In contrast, the organellar marker, FBP, was only detected in pellet fractions (Figure 2B). These results suggest that *L. major* MsrA is exclusively localized in the cytosol, as has been proposed for an isoform of *T. cruzi* MsrA [31].

To further investigate the role of LmMsrA, an *L. major* null mutant was generated using standard homologous recombination techniques in which the two chromosomal alleles encoding LmMsrA were replaced with drug resistance cassettes. A number of isolates were recovered after two rounds of selection. Deletion of the msrA gene and correct integration of bleomycin and puromycin resistance cassettes was confirmed using PCR (data not shown). Promastigote stages of these *L. major* Δ*msrA* null mutants displayed similar growth kinetics in standard culture medium as wild type *L. major* promastigotes (data not shown). These results suggest that *Leishmania msrA* is not essential during cultivation in rich medium.

### LmMsrA is a Functional Methionine Sulfoxide Reductase, as Loss of *msrA* Leads to Higher Levels of Methionine Sulfoxide within the Parasite

**Figure 3:**
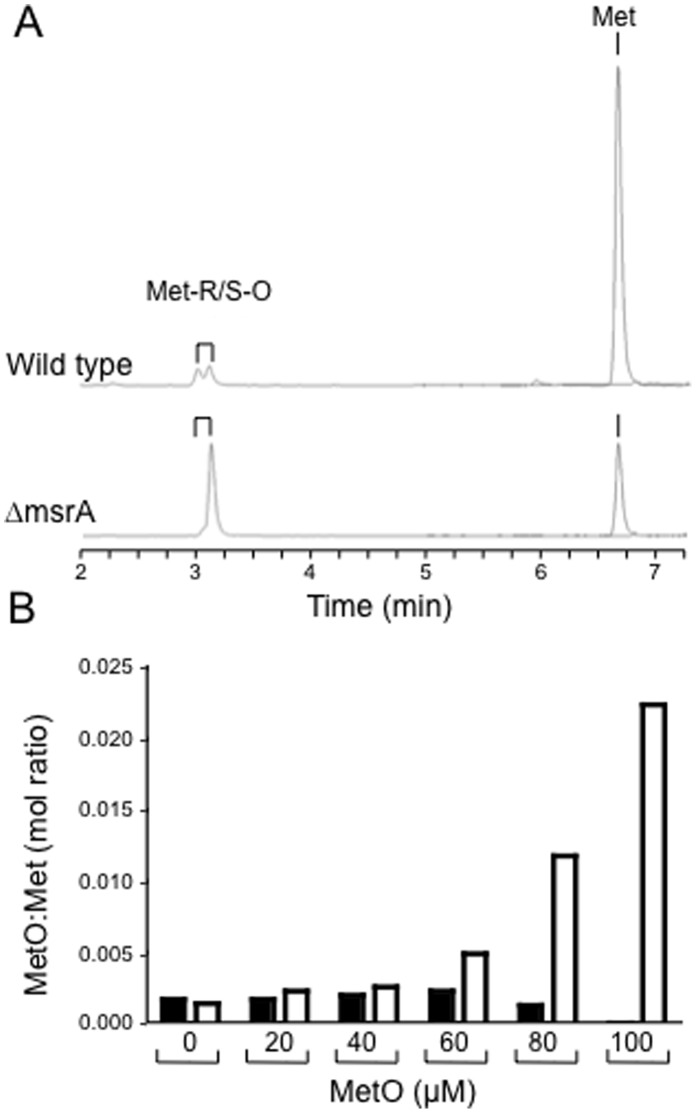
LC-MS quantification of intracellular MetO and Met levels in *L. major* grown in varying concentrations of Met and MetO. A. Abundance of free MetO (comprising a doublet of Met-S-O/Met-R-O isomers) and Met in total extracts of wild type (upper panel) and ΔmsrA (lower panel) promastigotes cultivated in media containing 80 µM MetO and 20 µM Met. B. Ratio of MetO to Met levels in *L. major* wild type (black columns) and Δ*msrA* (white columns) grown in varying concentrations of MetO.

The *E. coli* and *Salmonella* MsrA enzymes can reduce both free- and protein-bound Met-S-O back to methionine [25], [27]. In order to establish if LmMsrA has similar enzymatic activity, despite the alteration in the catalytic site, *L. major* wild type and Δ*msrA* strains were cultured in the presence of varying concentrations of Met and MetO (final concentration 100 µM). *Leishmania* are methionine auxotrophs and are therefore dependent on uptake of methionine for growth. We reasoned that wild type, but not Δ*msrA* mutant promastigotes would be able to reduce any MetO taken up by the methionine transporters. Parasites were grown in minimal media containing Met and/or MetO for five days and polar metabolites extracted and analyzed by liquid chromatography-mass spectrometry. Both strains contained very low levels of MetO (0.1–0.2 percent of Met levels) when grown in the absence of exogenously added MetO (Figure 3C). Wild type *L. major* promastigotes maintained low intracellular levels of MetO even when cultivated in high concentrations of MetO (>50 µM) (Figure 3B). In contrast, intracellular levels of MetO in *L. major* Δ*msrA* promastigotes increased as the MetO concentration in the media was increased. Specifically, ΔmsrA promastigotes cultured in the presence of 100% MetO had 10-fold higher levels of MetO than wild type parasites cultured under the same conditions (Figure 3B). Significantly, only one of the two MetO isomers identified in the LC-MS analysis was elevated in the MetO-treated parasites (Figure 3A, lower panel). This finding is consistent with the observation that MsrA enzymes typically display a strong preference for the Met-S-O isomer and have minimal activity against the Met-R-O isomer. The low levels of the second isomer in both the wild type and Δ*msrA* parasites likely reflects the continued expression of MsrB and/or MsrC enzymes in both parasites lines. The latter has been shown to reduce free Met-R-O [25], [26]. Collectively, these results indicate that LmMsrA is a functional methionine reductase A.

### Deletion of *msrA* Leads to a Greater Sensitivity to Oxidative Stress *in vitro*

**Figure 4:**
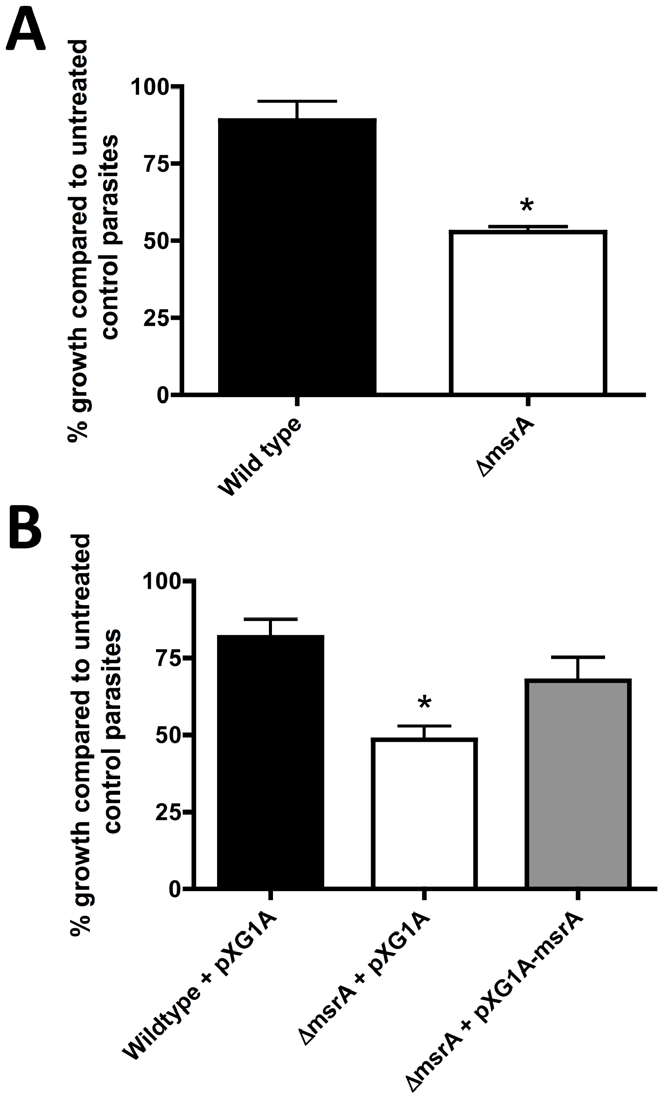
Recovery of *L. major* parasites following exposure to hydrogen peroxide treatment. Growth is expressed as a percentage of the growth of untreated control parasites (for each cell line) 3 days following H_2_O_2_ removal, as a measure of cell viability following H_2_O_2_ treatment. Error bars represent S.E.M. of three experiments. A**.** *L. major* Δ*msrA* is more susceptible to H_2_O_2_ treatment, with a significant decrease in cell viability compared to *L. major* wild type (*P = *0.02, unpaired t-test). B. *L. major* Δ*msrA*+pXG1A is also more susceptible to H_2_O_2_ treatment, with a significant decrease in cell viability compared to *L. major*+pXG1A (*P = *0.01, unpaired t-test). In contrast, no significant decrease in cell viability is observed for the complemented mutant strain Δ*msrA*+pXG1A-*msrA* when compared to *L. major*+pXG1A (*P = *0.22, unpaired t-test).

Loss of MsrA results in greater susceptibility to oxidative stresses in a number of organisms [27], [38]. Conversely, overexpression of *msrA* in *T. cruzi* increased resistance to exogenous oxidative stress, such as hydrogen peroxide treatment [31]. We therefore examined whether the Δ*msrA* mutant exhibited increased sensitivity to exogenous oxidative stress. Parasites were treated with 0.2 µM H_2_O_2_ for 24 hours and parasite viability measured by following parasite outgrowth in fresh media. Wild type *L. major* exhibited minimal growth retardation after H_2_O_2_ treatment compared to untreated, control parasites (Figure 4A). In contrast, growth of *L. major* Δ*msrA* was significantly reduced (∼50% of untreated parasites) following H_2_O_2_ treatment, demonstrating a heightened sensitivity of parasites to exogenous oxidative stress in the absence of LmMsrA.

To confirm that the observed difference was due to the absence of the LmMsrA, the *L. major* Δ*msrA* mutant was transfected with a pXG1A plasmid encoding the *msrA* gene. Transfection with pXG1A-msrA largely restored resistance to exogenous H_2_O_2_, as the viability of the complemented strain after three days of exposure to H_2_O_2_ was not significantly different from the wild type parasites (Figure 4A). In contrast, transfection of the Δ*msrA* mutant with the empty pXG1A plasmid did not increase the resistance of the mutant to oxidative stress (Figure 4B). These results provide direct evidence for *msrA* having a role in parasite resistance to oxidative stress.

### Loss of *msrA* Results in Decreased Replication within Macrophages *in vitro*, but is Not Required for Pathogenesis in the Mouse Model of Cutaneous Leishmaniasis

**Figure 5:**
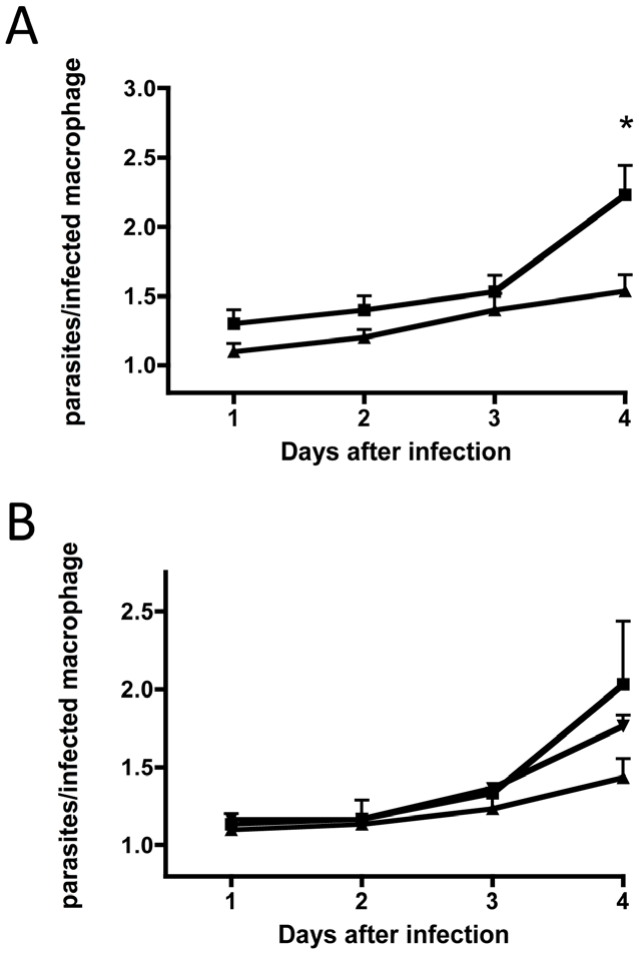
Infection of RAW 264.7 murine macrophages with *L. major* promastigotes. Data is presented as the number of parasites per infected macrophage, as observed by microscopy. Error bars represent S.E.M. from three independent experiments performed in triplicate. A. Macrophages infected with *L. major* wild type (squares) or *L. major* Δ*msrA* (triangles). A significant decrease in parasite numbers is observed at day 4 for *L. major* Δ*msrA* (*P = *0.01, unpaired t-test). B. Macrophages infected with *L. major*+pXG1A (squares), *L. major* Δ*msrA*+pXG1A (triangles) or *L. major* Δ*msrA*+pXG1A-*msrA* (inverted triangles).

*L. major* parasites proliferate within the mature phagolysosome of mammalian macrophages, where they are likely to be exposed to high levels of reactive oxygen species. Although *L. major* has a number of mechanisms to avoid the oxidative defense mechanisms of the host, these parasites are unable to completely inhibit the macrophage oxidative burst during invasion and subsequent proliferative phases [7] raising the possibility that LmMsrA may be important for parasite proliferation within this compartment. Murine RAW 264.7 macrophages were infected with *L. major* wild type and Δ*msrA* stationary phase promastigotes and parasite proliferation assessed over 4 days. Both wild type and Δ*msrA* parasites exhibited similar rates of macrophage invasion and the number of macrophages infected with both strains remained similar over 4 days (data not shown). However, proliferation of *L. major* Δ*msrA* strain was markedly attenuated compared to *L. major* wild type parasites after four days (Figure 5A). A similar result was observed when macrophages were infected with *L. major* wild type+pXG1A and *L. major* Δ*msrA*+pXG1A, with the number of *L. major* wild type+pXG1A parasites again approaching twice that of *L. major* Δ*msrA*+pXG1A (Figure 5B). In contrast, growth of the complemented mutant *L. major* Δ*msrA*+pXG1A-*msrA* more closely resembled that of *L. major* wild type+pXG1A (Figure 5B), indicating that the observed defect in intracellular growth was due to loss of *msrA*.

**Figure 6:**
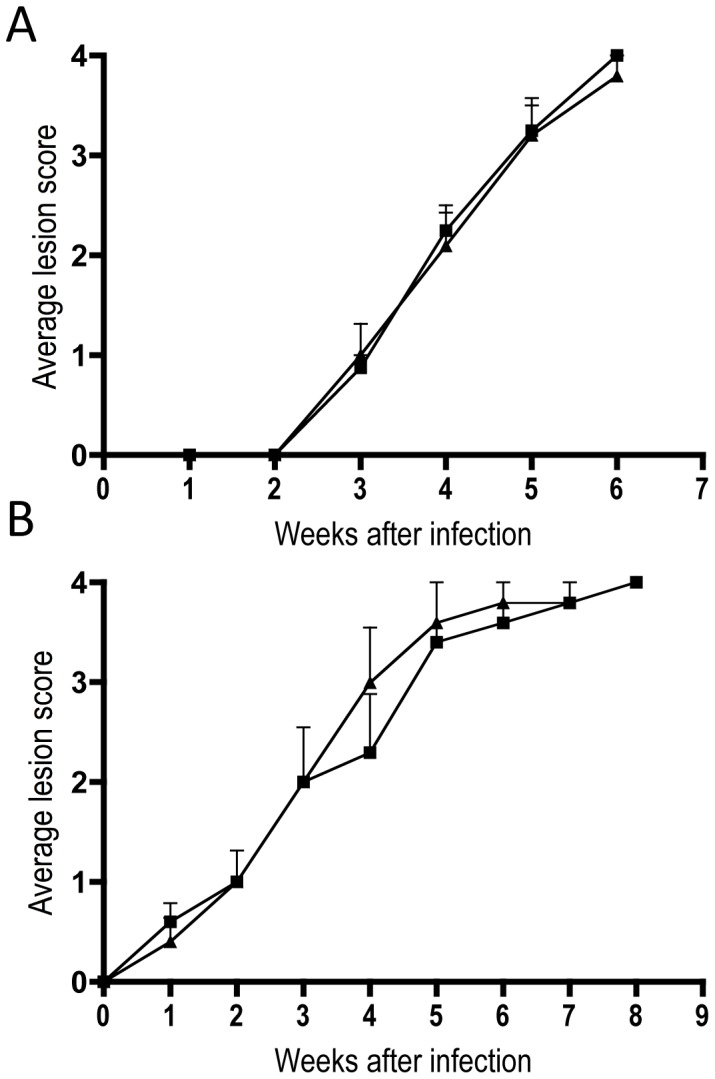
Subcutaneous infection of susceptible BALB/c mice with either promastigote (A) or amastigote (B) forms of *L. major*. Mice were infected with either wild type (squares) or Δ*msrA* (triangles) parasites and lesion scores monitored weekly. Error bars represent S.E.M. (*n = *5).

To investigate whether this replicative defect was important in the pathogenesis of the parasites *in vivo*, susceptible BALB/c mice were subcutaneously infected with either stationary-phase promastigotes or lesion-derived amastigotes. Following infection, the size of the inflammatory lesion was assessed weekly. Unexpectedly, no difference in average lesion size was observed for *L. major* Δ*msrA* compared to *L. major* wild type, when either promastigote or amastigote stages were used to initiate infections (Figures 6A and B). These studies suggest that additional oxidative stress protective mechanisms are activated in *in vivo* parasite stages, compared to amastigotes in *ex vivo*-infected macrophages, which can compensate for loss of MsrA.

## Discussion

*Leishmania* parasites have developed a number of strategies for dealing with reactive oxygen and nitrogen species generated within the host macrophage despite lacking a number of key antioxidant enzymes, such as catalase and glutathione peroxidase. These strategies can be broadly grouped into avoidance of the host antioxidant response, neutralization of host generated reactive oxygen and nitrogen species and repair of oxidatively damaged proteins. While the first two lines of oxidative defense have been intensively studied [6], little is known about the protein repair mechanisms of *Leishmania*. Proteinogenic methionine residues are highly susceptible to oxidation and in this study we have investigated the potential role of a putative methionine sulfoxide reductase A in *Leishmania* pathogenesis. *Leishmania* MsrA is encoded by a single gene that is well conserved, both in terms of sequence homology (76–90%) and synteny across the *Leishmania* genus. MsrA gene homologues are also present in the genomes of other trypanosomatid parasites and exhibit ∼40% sequence homology to human and *E. coli* MsrA. The amino acid sequences of *L. major* MsrA is notable in containing a tyrosine residue instead of phenylalanine in an otherwise highly conserved sequence in the catalytic site. This change was conserved in all species of *Leishmania* and has not been reported in any other organism. Mutation of this Phe residue to Ala or His in the bacterial MsrA proteins is associated with a complete loss of enzymatic activity [33]. The Phe to Tyr change observed in the *Leishmania* sequences is more conservative, but nonetheless results in addition of an extra hydroxyl group and increased polarity in the catalytic motif. Thus it was important to establish if LmMsrA was functional. An *L. major* strain lacking both alleles of *msrA* was readily generated indicating that LmMsrA is not essential for growth under standard culture conditions. When wild type and Δ*msrA* mutant parasites were cultivated in the presence of exogenous methionine sulfoxide (MetO), the mutant parasites, but not the wild type parasites, accumulated significant levels of MetO, indicating that MsrA is functional. The Δ*msrA* parasites accumulated a single isomer of MetO providing further evidence that the *msrA* gene encodes a classic MsrA with selectivity for the Met-S-O isomer, as well as indicating the presence of other methionine reductases with selectivity for the Met-R-O isomer. Interestingly, neither wild type nor Δ*msrA* parasites were able to grow in methionine-free medium supplemented with MetO (data not shown). The rate of reduction of MetO may therefore be insufficient to supply the nutritional needs of these parasites and/or the rapid incorporation of MetO into other metabolic intermediates or proteins may result in growth inhibition.

The *L. major* MsrA protein lacked any recognizable organelle-targeting signals indicating that it is primarily or exclusively localized in the cytosol. This localization was confirmed by localization of an MsrA-GFP-fusion protein in live promastigotes by fluorescence microscopy and by subcellular fractionation. The MsrA proteins of *T. cruzi* and *S. cerevisiae* have also been shown to have a cytosolic localization [31], [39]. In contrast, two isoforms of MsrA are expressed in mammalian cells, which are targeted to the cytosol and the mitochondrion, respectively [37], [40]. Whether other methionine reductases in *Leishmania* and other trypanosomatids are targeted to the mitochondrion remains to be defined.

Deletion of the *L. major msrA* gene resulted in increased promastigote sensitivity to external oxidative stress (H_2_O_2_). These findings are consistent with a recent report showing that overexpression of MsrA in *T. cruzi* epimastigotes results in increased resistance to oxidative stress [31]. The mutant line also exhibited a reduced capacity to replicate as amastigotes in murine macrophages, consistent with a number of expression profiling studies indicating that MsrA is constitutively expressed in both major developmental stages [41], [42]. Collectively, these findings suggest that MsrA has a significant role in repairing oxidatively damaged parasite proteins and/or reversing the oxidation of potentially limiting levels of methionine in intracellular stages [43]. Unexpectedly, deletion of *L. major msrA* had no discernable impact on lesion development in the highly susceptible BALB/c mouse model of cutaneous leishmaniasis. *L. major* infection in BALB/c induces a strong anti-inflammatory Th2 response, with concomitant suppression of classic activation and induction of the oxidative burst in infected macrophages (reviewed in [44]). Under these conditions, MsrA mediated repair of proteins may not be essential for parasite proliferation. Alternatively, the reduced virulence of the *L. major* Δ*msrA* mutant in *ex vivo* infected macrophages but not in the BALB/c murine models, could reflect the up-regulation of additional parasite stress responses in the mammalian host. These could include increased expression of heat shock proteins, proteasome activity, lysosomal enzymes or autophagy [45]–[47], all of which may have a role in either preventing protein miss-folding or removing damaged proteins and have been shown to be essential for *Leishmania* amastigote survival *in vivo*.

The absence of a strong loss of virulence phenotype in the Δ*msrA* mutant may also reflect redundancy in methionine protein repair mechanisms. Specifically, the *L. major* genome contains genes for putative MsrB (*LmjF28_2660*) and MsrC (*LmjF23_1460*) enzymes [48]. While MsrA is responsible for reversing the oxidation of the free and proteinogenic Met-S-O epimer, MsrB and MsrC are primarily responsible for repairing the Met-R-O epimer in damaged proteins and free Met-R-O, respectively [27], [49], [50]. MsrA has been shown to be important for the virulence of several bacterial pathogens, including *Helicobacter pylori* [51], *Salmonella typhimurium* [27], *Mycobacteria genitalium* [29] and *M. smegmatis* [34], [35]. In *S*. *typhimurium* and *M. smegmatis*, the *msrB* enzyme appeared to play a limited role in resistance to oxidative stress as deletion of the corresponding genes had little effect on the intracellular growth of these pathogens [27], [34], [35]. However, deletion of both *msrB* and *msrC* genes in *S. typhimurium* resulted in a severe attenuation of virulence indicating that these enzymes may have redundant roles in repairing the Met-R-O epimer [27]. In considering the role(s) of MsrA/B/C in microbial pathogenesis, it is important to note that these proteins may become essential under certain growth conditions or during specific stages of infection. For example, expression of MsrA is required for the proper functioning of extracellular adhesins [29], [52], [53], motility [54] and biofilm production [53] in several bacterial pathogens, reflecting the oxidative sensitivity of specific proteins involved in pathogenesis. The reduced virulence of the *L. major* Δ*msrA* mutant in *ex vivo*-infected macrophages could therefore reflect a dependency on one or more oxidatively sensitive proteins that are not required for infection *in vivo*.

In conclusion, this is the first study to examine the role of MsrA, or indeed any oxidative damage repair mechanisms, in human pathogenic protozoon parasites. We show that LmMsrA confers protection against exogenous oxidative stress, and is required for normal growth in macrophages. Surprisingly, we find that MsrA is not essential for lesion development in mice. In future studies, it will be of interest to delineate the potentially redundant roles of MsrB and MsrC in *Leishmania* infectivity.

## Materials and Methods

### Ethics Statement

Use of mice was approved by the Institutional Animal Care and Use Committee of the University of Melbourne (ethics number 0811011.1). All animal experiments were performed in accordance with the Australian National Health Medical Research council (Australian code of practice for the care and use of animals for scientific purposes, 7^th^ Edition, 2004, ISBN: 1864962658).

### Bioinformatic Analyses

BLAST [32] searching of the *Leishmania major* strain Friedlin genome [48] was used to identify putative Msr enzymes. Protein sequence alignments were performed using ClustalW [55], [56] and edited using JalView software [57].

### Parasite Strains and Culture Conditions

*L. major* substrain MHOM/SU/73/5-ASKH was used to create all mutant and transfected lines. Parasites were routinely cultured as axenic promastigotes in Medium-199 (M199, Gibco, Invitrogen, Australia) supplemented with 10% heat-inactivated foetal bovine serum (FBS, Invitrogen) at 27°C or, prior to infection experiments, in SDM-79 medium supplemented with 10% FBS. G418 (Invitrogen, 100 µg/mL) was used to maintain selection pressure on parasites transfected with pXG1A plasmids, while puromycin (Invitrogen, 20 µg/mL) and/or bleocin (Calbiochem, 10 µg/mL) were used to select transformants during mutagenesis. Lesion amastigotes used in infection studies were prepared by disrupting murine lesions (diameter 5–10 mm) by passage through a stainless steel sieve and repeated passage through a 27 G needle to lyse macrophages and release parasites. Cell debris was removed by centrifugation (50*×g*, 10 min, 4°C) and amastigotes in the supernatant collected by a second centrifugation step (2000*×g*, 10 min, 4°C). Amastigotes were washed in sterile PBS and counted using a haemocytometer prior to use in infection studies.

### Genetic Manipulation of *L. major*

*L. major* Δ*msrA* was created via sequential homologous gene replacement in a manner similar to that previously described [58], [59]. Briefly, a 935 bp 5′ untranslated region (UTR) containing a 5′ HindIII site and a 3′ BamHI/EcoRI*/*linker region was amplified using primers 5′-CCCAAGCTT CGTACGTGCGCATTCTTCGCC -3′ and 5′-CCGCTGGGATCCGAATTCGACGGGTGGGTGGGTG-3′, and a 980 bp 3′ UTR region containing a 5′ BamHI*/*EcoRI*/*linker region and a 3′ HindIII site was amplified using primers 5′-GAATTCGGATCCCAGCGGGCGGGTCGCTGCTGTCAC-3′ and 5′- ATAAGAATGCGGCCGCCAGGCACGCGCTGCC-3′. An overlap PCR was then performed using these PCR products as template, and the resultant product cloned into the HindIII/NotI sites of the pBluescript II SK vector (Stratagene, CA, USA). Puromycin or bleocin resistance cassettes were excised from either pXG-PAC or pXG-PHLEO [60] respectively using BamHI and EcoRI, and cloned into the engineered BamHI/EcoRI sites. Deletion mutant constructs were verified by restriction digest profiles and DNA sequencing. The PAC- and BLE-containing *msrA* targeting constructs were excised by HindII/NotI digest, gel purified and 5 µg of each sequentially electroporated into *L. major* as described previously [61]. Clonal transformants resistant to both puromycin and bleocin were selected and deletion of *msrA* confirmed via triplicate PCR (data not shown). To generate the pXG-*msrA* vector for complementation studies, full-length *msrA* gene was amplified by primers 5′-CTCCCGGGATGCCCGCACCTATCCGAGCC-3′ and 5′-CGGGATCCCTAAAAGTACAAGCGGTGGAGC-3′ and cloned into the SmaI/BamHI sites of the pXG1A vector. To create the MsrA-GFP fusion protein, full length *msrA* was amplified using primers 5′-CCCGGATCCACCATGCCCGCACCTATCCCGAG-3′ and 5′-CGATATCAAAGTACAAGCGGTGCGAGCAG-3′ and cloned into the BamHI and EcoRV sites of pXG-GFP^+^ [62]. The resulting pXG-derived constructs were confirmed via DNA sequencing and electroporated into *L. major* as previously described [61].

### Detection of LmMsrA-GFP Using Immunoblotting and Microscopy

Subcellular fractionation of parasites was performed as described previously [63]. Briefly, log phase parasites were harvested via centrifugation, washed in PBS and resuspended in hypotonic lysis buffer (1 mM NaHEPES-NaOH, pH 7.4, 1 mM DTT and 2 mM EGTA, protease inhibitor cocktail (Complete-EDTA free, Roche, Germany), 10 min 0°C) and cell lysis achieved by passage through a 27 G syringe needle. The lysis buffer was adjusted to 50 mM NaHEPES-NaOH, pH7.4 and cell debris was removed by low speed centrifugation (500*×g*, 15 min, 0°C), and the supernatant subjected to sequential centrifugation at 10,000*×g*, and 100,000*×g* (30 min, 4°C). The pellet and supernatant fractions were analyzed by SDS-PAGE and MsrA-GFP fusion protein detected using the anti-GFP antibody (clones 7.1 and 13.1, Roche, Germany) at 1∶1000 dilution with the signal detected using standard chemiluminescent techniques. The membrane was then probed with a antibody to the organellar marker, fructose-1,6-bisphosphatase (FBP) [59] (diluted 1∶100). For microscopy studies, live cells were immobilized on poly-L-lysine coated coverslips and visualized using a Zeiss Axioplan 2 epifluorescence microscope. Images were obtained using an AxiCam MRm camera and AXIOVISION 4.3 software.

### LC-MS Measurement of Methionine/Methionine Sulfoxide

*L. major* promastigotes were cultured in completely defined media [64] containing 0–100 µM methionine (Met, Sigma, USA) and 0–100 µM methionine sulfoxide (MetO, MP Biomedicals, Australia) at a final concentration of 100 µM. Cells were incubated for 5 days and metabolites extracted as previously described with rapid metabolic quenching [65]. Briefly, parasite metabolism was quenched by rapid cooling to 0°C in an ethanol/dry-ice slurry and parasites rapidly harvested by centrifugation (3,200*×g*, 10 min, 0°C). The pellet was washed in cold PBS and parasites lysed in chloroform:methanol:water (1∶3:1, v/v, 250 µL) with sonication and incubation on ice for 15 minutes. Insoluble material was removed by centrifugation (10,000*×g*, 5 min, 0°C) and water (100 µL) added to generate a biphasic mixture after centrifugation (10,000 *g*, 5 min, 0°C). The upper, aqueous phase was dried under nitrogen in 250 µL glass vials and amino compounds derivatised with aminoquinolyl-N-hydroxycuccinimidyl carbamate (AQC) reagent [66] prior to liquid chromatography-mass spectrometry (LC-MS) analysis [66]. Multiple reaction monitoring was used to detect methionine and methionine-S/R-sulfoxide peaks using m/z 320/171 and m/z 336/171 transitions, respectively.

### Induction of Oxidative Stress *in vitro*

*L. major* promastigotes (2×10^5^) were exposed to fresh H_2_O_2_ (20 µM) for 24 h, then harvested by centrifugation and resuspended into fresh M199 media. Untreated control parasites were included for each strain tested. Parasites were incubated at 27°C for 5 days and viable numbers assessed every 24 h by counting using a haemocytometer, as an indirect measure of the viable number of parasites surviving H_2_O_2_ treatment.

### Macrophage and Mouse Infections

Infection of RAW 264.7 murine macrophage cells was performed as described previously [67]. Briefly, macrophages growing on coverslips were infected at a multiplicity of infection (MOI) of 10 with stationary phase promastigotes and incubated at 33°C in RPMI medium supplemented with 10% FBS and 5% CO_2_. Macrophages were washed after 4 h to remove extracellular parasites and coverslips fixed with 4% paraformaldehyde every 24 hours for four days. Intracellular parasites were stained with Hoechst and triplicate coverslips were counted at each time point, with a minimum of 100 macrophages per coverslip, to determine parasite infection levels. Three independent infections were performed in triplicate. To assess virulence in mice, 6–8 week old (age-matched) female susceptible BALB/c mice were injected subcutaneously at the tail base with either 5×10^5^ amastigotes or 1×10^6^ stationary phase promastigotes. Lesion size was assessed weekly and scored 0–4, as described previously [68]. All parasite cell lines had been passaged previously in mice to ensure no loss of virulence unrelated to the known genetic mutations, and parasites re-isolated from mice as described previously [61].
